# Endocrinology specialty service for inpatients: an unmet growing need

**DOI:** 10.1186/s12913-023-09134-y

**Published:** 2023-02-10

**Authors:** Esther Osher, Naomi Even Zohar, Michal Yacobi-Bach, Dror Cantrell, Merav Serebro, Yael Sofer, Yona Greenman, Karen Tordjman, Naftali Stern

**Affiliations:** 1 Institute of Endocrinology, Metabolism and Hypertension, Tel Aviv-Sourasky Medical Center, 6 Weizmann Street, Tel Aviv, 6423906 Israel; 2 Sackler Faculty of Medicine, Tel Aviv University, Tel Aviv, Israel; 3 Department of Internal Medicine C, Shamir Medical Center, Zerifin, Israel

**Keywords:** Endocrine consult service, Inpatient, Outpatient

## Abstract

**Background:**

There is recent concern regarding the documented mismatch between demand and supply, vis-à-vis the growing need for trained endocrinologists unmet by parallel rise in the world workforce of endocrinologist. Due to the increasing complexity of disease in inpatients, in recent years we have experienced a growing demand for inpatient endocrine consults. Surprisingly, the need for the endocrinology subspecialty in the overall care of inpatients in the current setting of general hospitals has received little attention.

**Methods:**

A retrospective analysis of endocrine consult service based on solicited consults carried out during 3 consecutive months.

**Results:**

During 3 months, there were 767 consults, comprised of 156 diabetes referrals and 611 endocrine/metabolic consult requests. The 611 "non-glucocentric" consult requests were related to 295 inpatients (2.1 ± 2.7 consults/patient). Mean patient age was 58.9 ± .18 years (range 21–92), with some F/M preponderance (58/42%). Requests for endocrine consults were evenly distributed (49.8%, 50.2%) between internal medicine and surgery wards. Case distribution was as follows: thyroid 45.4%, calcium & bone 11.5%, pituitary 12%, adrenal 10% and all others 8.1–0.7%. The mean response time was 4.4 ± 2.7 h. The consults had a discernible effect on the patients' disease management in 60% of the patients. Of these, the consults modified the hospital treatment in 74%, the discharge treatment recommendations in 19% and the diagnosis in 7%.

**Conclusion:**

At a large medical center, endocrine consults were requested for ~ 3.3% of all admitted inpatients. The endocrine consults modified pre-consult diagnosis or treatment in ~ 60% of the cases. Contrary to its common image as an exclusively outpatient-based subspecialty, endocrinology practiced by specialists and endocrine trainees has a notable role in the daily care of inpatients admitted to a referral general hospital.

## Introduction

The educational need for the involvement of endocrinologists and endocrine fellows in the care of hospitalized patients has been highlighted by a recent update of the requirements for training in endocrinology by the European Union of Medical Specialists [1]. According to this expert consensus document, hospitals comprise the organizations, which are sufficiently enriched with the supportive environment needed for training in terms of highly specialized teams and sufficiently intensive interactions with other subspecialties, equipment and laboratories [1]. On the patients' receiving end, the input offered by the endocrine subspecialty may favorably affect each phase of the hospitalization in many cases, from admission via the emergency department, in-hospital diagnosis and treatment, culminating in discharge preparation and instructions. However, the actual role of endocrinology in the current care of hospitalized subjects has received little attention and was not targeted as an independent subject of study. Historically, this void has been aggravated by the fact that with the exception of some dedicated clinical research facilities or subspecialty–based wards in a few European countries, inpatient endocrine wards are nearly extinct and the bulk of endocrine practice has shifted to the outpatient setting, mostly in the community.

Because low level of awareness of the role of endocrinology in inpatient care may lead to improper allocation of personnel in hospital endocrinology centers, we set out, in the present report, to assess 1) the endocrine patient load, disease profile, and service availability during hospital admissions; 2) the impact of the endocrine consults on patient care during in-hospital stay. The general setting was our center, which is a tertiary referral, 1400 bed hospital, with a well-developed endocrine group.

## Methods

### Data collection

This study was approved by the Review Board at our center. We carried out a retrospective analysis of the adult endocrine consult service based on solicited consults carried out during 3 consecutive months (April 1 through June 30, 2015) at the Medical Center. All consecutive consults placed during that period were reviewed and the parameters of interest extracted.

### Mode of operation of the endocrine consult service

The consult service operates via a computerized system such that request for consults, when are placed continuously by hospital staff, usually by residents or fellows from all adult wards, with the approval of attending physicians. Requests are submitted when a new endocrine diagnosis is under consideration or when changes in treatment are entertained and the team believes that input from the endocrine service would benefit the patient.

### Diabetes consult service

The diabetes unit is integrated into the Institute of Endocrinology and diabetes consults are provided by either the diabetes unit staff or the entire endocrine, staff as needed.

The endocrine consult service operates with a three-tier system comprised of a fellow, rotating senior endocrinologist in charge and endocrine subspecialists (e.g., neuroendocrine tumors). Eventually, the vast majority of requests for consultations originate in general medicine and surgery, specialized wards (such as cardiology, neurology, dermatology, neurosurgery, oncology, orthopedic/oncological surgery, head and neck surgery etc.), general and specialized intensive care units, as well as the emergency department. Finally, no financial restrictions/reimbursement considerations are applied with regard to the use of professional advice during hospitalization in this system, which is typical for a public hospital operating within a nationalized health care system.

### Data Analysis

The following parameters were evaluated: 1) number of consults/day or source (hospital unit) of the request; 2) number of consults per patient/admission during study period; 3) the response time of the consultation, from the placement of the request to the written endocrine report; 4) patients' age and gender; 5) reason for consultation, categorized by the endocrine disorder addressed; 6) overall disease severity, ranked by the first author's judgment (EO), using the patient's entire medical file as constructed during the hospitalization period. Electronic files include daily ward staff notes, laboratory and imaging reports, consults from other disciplines, nursing notes and admission and discharge reports. For the purpose of this analysis, the severity of illness during the consultation time was also assessed. Severity rank was adopted from published models and sets of criteria [2] and scored as follows: mild=1; moderate=2; severe=3 (Table 1a). 7) effect of the consult on diagnosis and treatment, using the following categories: change in diagnosis (recorded as 1), change in treatment during hospitalization days (recorded as [2] or change in the discharge treatment instructions, recorded as [3]. 8) effect of the consultation ranked according to the level of the documented change in the endocrine condition during hospitalization as assessed at the time of data analysis, categorized as follows: no change, deterioration; partial improvement in illness severity status; improvement in illness severity status. We only graded the changes that could be reasonably linked to consult- related actions. For example, if hypoadrenalism related to immune checkpoint modulators was corrected and stress doses were used, but the patient succumbed to ascending cholangitis with pseudomonas sepsis following chemotherapy, the deterioration was not ascribed to the consult.

Analysis was done by SPSS software, analysis included, descriptive measures frequencies, means, ranges, and medians and correlation using non parametric test spearman correlation test. Results are presented as means+/-SD.

## Results

**Fig. 1:**
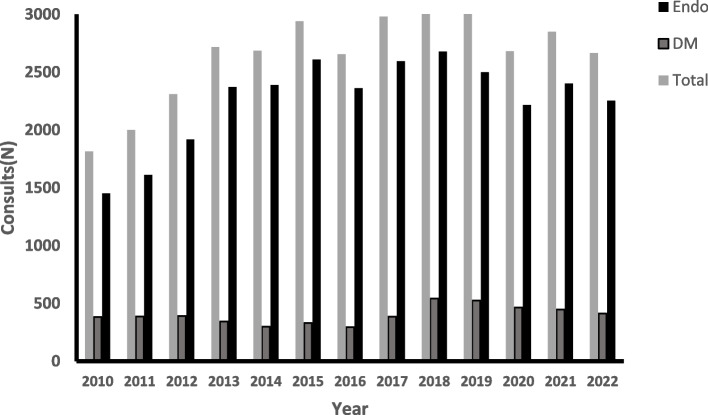
Number of endocrine inpatient consults in the years 2010–2022. The number of endocrine consults at our center showing, an increase from 1814 ~ in 2010 to ~ 2664consults in 2022

The overall trend in the number of total endocrine consults at our center is depicted in Figure 1, showing, in essence, an increase from ~1800 consults in 2010 to ~2700 consults in 2022(*p*<0.001). There was also an increase in the number of diabetes consults (which comprised ~15% of total consults), between 2010-2022 from~380 to 540(*p*<0.001). However, despite the increase in patient hospital admissions during COVID19 pandemic, there was no significant increase in the number of total consults between end of 2019 to 2022. Zooming on the 3 months surveyed here, there were 767 endocrine consults, which amounted to 8.4 requests/day, or 11.8 requests /day if only the 5 full working days of the week are considered. 156 of these requests (20.3%) were to assist in the control of glucose in patients with diabetes. Hence, there were 611 "non-glucocentric" consult requests, relating to 295 inpatients (on the average, ~2 consults/patient during the hospital stay).

In all, endocrine consults were requested and received for 0.013% of the adult subjects hospitalized at the center during the survey period, of which 0.66% centered on glucose control and 2.6% addressed endocrine/metabolic issues that did not mainly deal with glucose control. In the non-diabetes consults, mean patient age was 58.9±18yrs. (range 18-95), with some F/M preponderance (58 /42%).

**Fig. 2:**
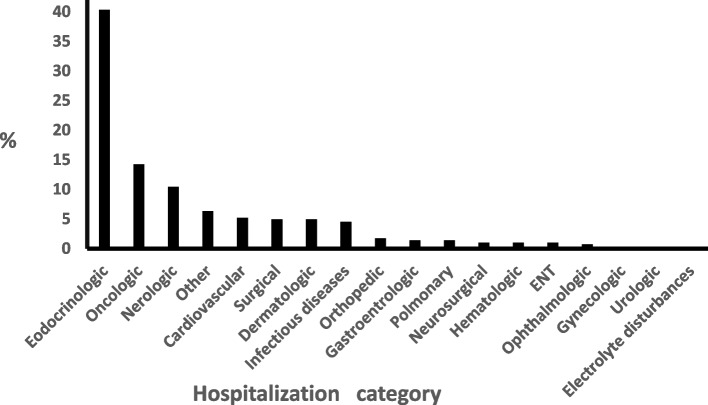
Distribution (%) of the primary causes for admission in the study cohort by medical categories. The primary reason for the acute admission in subjects for whom an endocrine consult was requested during hospitalization was endocrine in 40%, oncologic in 14%, neurologic in 10%, with the remaining third (36%) linked to variety of multiple other conditions

**Fig. 3:**
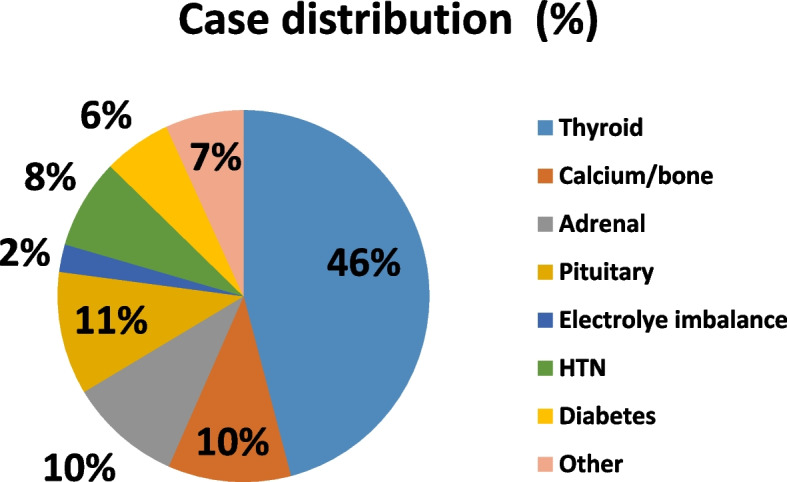
HTN = hypertension. Endocrine consults case distribution(%) The distribution of the underlying reason for requesting the endocrine consult was as follows: thyroid- 45.4%, calcium & bone-11.5%, pituitary-11.9%, adrenal-9.8% with all other fields accounting for 0.7–8.1% each. This sub-analysis is limited to the non-glucocentric consults only

Requests for endocrine consults were evenly distributed (49.8%, 50.2%) between internal medicine and surgery wards. With the exclusion of diabetes, the primary reason for the acute admission in subjects for whom an endocrine consult was requested during hospitalization was endocrine in 40%, oncologic in 14%, neurologic in 10%, with the remaining third (36%) linked to variety of multiple other conditions, each accounting for 0.3-6% of the admissions (Figure 2). The distribution of the underlying reason for requesting the endocrine 611 "non-glucocentric" consult was as follows: thyroid- 45.4%, calcium & bone-11.5%, pituitary-11.9%, adrenal-9.8% with all other fields accounting for 0.7-8.1% each (Figure 3). Finally, the distribution of the severity of disease during the time of actual consultation was as follows: grade 1(mild or less); in ~80%, grade2 (moderate) in ~19% and severe in ~2%.

Overall, there were 2.1±2.7 consultations per patient and the mean response time was 4.4±2.7h. There was a modest correlation between disease severity and the number of consults requested per patient (R=0.3; *P*<0.05). There was also a modest correlation between the number of per patient consultations and the estimated effect of the intervention as defined above (R=0.33; *P*<0.05).

Finally, we assessed, in each case, whether or not the management in the course of the patient's hospitalization was modified by the suggestions offered in the endocrine consults. In all, the interventions initiated by the consults led to a change in the endocrine care in 60 % of the patients seen. Of this subset of patients, a change in treatment during the hospitalization took place in 74 %; a change in their discharge treatment instructions was recorded in 19%; and a change in diagnosis occurred in in 7% (Table2).

**Table 1:** Grading the severity of disease during the consultation

	Grade 1	Grade 2	Grade 3
	**Mild or less**	**Moderate**	**High -Catastrophic**
Complications of the principal condition	mild or less	Moderate	High
Dependency (on the help of others for daily activities of life such as washing, eating) due to illness	None	Partial	Complete
Risk for morbidity /short mortality	Mild or less	Moderate	High

Any item on the second column would grade patient's status as "moderate; any item in the third column (high severity) would grade the patient's status as "severe" (adopted from reference 9)

**Table 2:** Consult change in the endocrine care in 176 patient, 60% of study cohort

Type of change	%
**Change in treatment during hospitalization**	74% (*n* = 130)
**Change in treatment instructions on discharge**	19% (*n* = 33)
**Change in diagnosis**	7%(*n* = 12)

The effect of the consultation was also ranked by to the level of change in the endocrine condition during hospitalization. As judged at the time of data analysis, no change/no improvement in the condition for which the consult was requested was seen in 37 % of the patients; partial improvement in 50 % whereas a clear improvement was noted in 13%. We did not identify cases in which consult-related intervention result in deterioration in patients' clinical status. This assessment is admittedly subjective as we did not have a "case control" setup.

## Discussion

Notwithstanding that nearly all major endocrine discoveries were made in hospital-based endocrinology centers, endocrinology/metabolism is now generally viewed as an "outpatient subspecialty". This most likely reflects the chronic nature of some of the commonest endocrine diseases such as hypothyroidism, diabetes, osteoporosis and obesity and the shrinkage of hospital endocrinology units. In 1998, describing the profile of the inpatient endocrine activity in the Mayo Clinic, Brennan et al [3] stated that "the nationally experienced decline in both hospital admission rates and length of stay has resulted in a reduction in the number of primary endocrine services at Mayo hospitals from three in 1980 to one currently". We were able to identify only a single previous report, in an abstract form, that formally quantified current endocrine workload in the setting of a general hospital. [4]. In all, the inpatient endocrine service at University Hospitals Birmingham NHSFT (IES@UHB) during 2010-2105 handled 2,817 inpatient referrals, amounting to an annual average of 470 cases. Importantly, referral volume grew at an average annual rate of 49.2% year-on-year, from 127 in 2010 to 885 in 2015, which is reminiscent of the trend at our institution, albeit with a smaller case load and a steeper annual increment. The trend was observed both in endocrine and diabetes consults, that comprised~15% of total consults. Interestingly at the COVID -19 pandemic period, there was no significant change in consult number of either endocrine or diabetes. It is possible that the extreme work overload secondary to the acute rise in critically ill, respirated patients, reduced the medical staff's time to communicate with consulting services which were limited to the most acutely critical dilemmas.

A report in 2008 indicated that nearly one in five adult patients admitted to a large general hospital had unrecognized probable diabetes, based on elevated HbA1c levels [5]. In an editorial published in 2011, Toledo and Stewart strongly objected to the possibility that primary care providers independently manage patients with diabetes, osteoporosis and thyroid disease [6]. Their key point was that endocrine care in times of explosion in the number of procedures, devices and particularly diabetes and osteoporosis drugs, often used in combination was too complex to circumvent professional endocrine advice. Ten years later, with the ongoing increase in life expectancy, gradual introduction of molecular and genetic testing and the advent of many oncology drugs with endocrine sequels [7] this has become even more complicated.

In a multi-authored e-handbook of inpatient endocrinology [8], Garg et al covered several dozens of specific emergency endocrine inpatient dilemmas. These critical instances take place on the background of the high prevalence of endocrinopathies in the general population. Golden et al [9] reported more than 10 endocrine/metabolic disorders whose prevalence in US adults exceeded 5 % including diabetes mellitus, impaired fasting glycaemia, impaired glucose tolerance, obesity, osteoporosis, osteopenia, vitamin D deficiency, erectile dysfunction, lipid disorders and autoimmune thyroiditis. Childhood cancer survivors were at increased long-term risk for diseases requiring inpatient treatment decades after their initial and 11% of these cases were due to endocrine disorders [10]. Endocrine disorders, then, afflict a sizable part of the population and, owing to clustering of conditions (e.g., obesity, diabetes, hypogonadism), many subjects would present with several endocrinopathies. Consequently, many hospital admissions not primarily related to endocrine disorders, such as ICU admissions [11], systemic or local infection, non-endocrine cancer or fractures necessitate reassessment and changes in the pre-admission treatment of the background endocrine conditions. Additionally, newly diagnosed endocrine disorders such as those elicited by immune checkpoint modifiers (e.g., thyroiditis) may emerge during, or complicate the course of hospital admissions. Mild hyponatremia (Na OF 130–135 mmol/L) is seen in up to 30% of hospitalized patients [12], whereas moderate to severe hyponatremia (Na+] <130) has been reported in 7% of inpatients [13]. In a prospective study of patients with syndrome of inappropriate anti diuretic hormone secretion (SIADH) who are often undertreated and mostly discharged with persistent hyponatremia, the involvement of endocrinologists improved the required time for correction of hyponatremia and shortened length of hospitalization [14]. Even if endocrine consultation is requested in a small fraction of such cases, the cumulative burden would appear significant. With the increasing fraction of older subjects in the general population, a disproportional rise in their hospital admissions associated age-related endocrinopathies and metabolic derangements are on the rise, among which previously undiagnosed diabetes is particularly common.

This manuscript describes the endocrine consultation service at our medical center, a 1400 bed tertiary referral center. Since consults were requested for ~1/33 (3.6) of admitted patients, the importance of providing an inpatient endocrine consultation service is almost self-evident. Keeping in mind the much higher rate of endocrine disease in the general population [9] and that hyponatremia alone afflicts nearly one third of inpatients [12], it is obvious that a marked degree of selectivity and restraint was applied by the hospital's staff in the use of requests for endocrine consultation. For example, if, as reported by Golden et al [9], the prevalence of hypo- and hyperthyroidism in the general population is ~6%, and requests for consults on thyroid disorders in at our center amounted to ~1.17% (2.6%X0.45) of the patients admitted during the study period, the advice of an endocrinology was asked in only 1/5 of the patients with known thyroid disease. A similarly calculated rate for hyponatremia, as one additional example, would have yielded a much lower rate of requests per hyponatremia cases. It is likely, but impossible to determine with certainty in a retrospective analysis, that suspicion of endocrine etiology instigated the interest in the opinion of an endocrinologist.

It is notable that in about 40 % of the patients for whom endocrine consultation was requested, an endocrine disorder was the primary cause of admission. This highlights the difficulty in managing some patients with endocrine disease in the outpatient setting.

The involvement of an endocrinologist had a notable impact on the care in 60% of the cases. The most prevalent effect was change in treatment in hospital and out of hospital. In the absence of a comparator group, such as might be offered by a parallel hospital lacking an active endocrine service, it is impossible to assess the effect of the endocrine service on tangible measures such as hospital stay or the admission outcome. Still, missed or delayed diagnoses during the short time window of hospitalization may be not only detrimental to patients' health, but severely aggravate the burden of later health and financial cost owing to complications and disability. The results are therefore valuable in that they portray, for the first time, the nature of current hospital endocrine work using several quantitative and semi quantitative measures. At the least, this report provides a clear justification for further in-depth studies and policy changes.

Recent reports have provided important information on the positive impact of a specialized diabetes team on short term outcome measures in patients with diabetes such as length of in-hospital stay and the number diabetes-related prescriptions [15–17]. Here we focused on the role of a general endocrine inpatient service.

Our analysis may be directly relevant only for large public hospitals providing the full range of current medical care. Nevertheless, many of the clinical dilemmas leading to the consult types included in the present survey are most certainly encountered in smaller hospitals which are set up for extended hospitalization or connected to neighboring daycare specialty centers such as oncological services.

Finally, mid-to-large hospitals, particularly those with academic affiliation, have traditionally undertaken the mission of training endocrine fellows. The demand for endocrinologists is presently unmet and the gap is projected to widen even further [18]. Further, recent concerns that the gap is still growing, were abetted by an anticipated sequel of this gap, a projected growing shortage in endocrine mentors and educators [19, 20]. To date, the latter could be bred only in the setting of strong endocrine hospital services.

In summary, this report underscores the important role of endocrinology in the hospital setting as an expanding aspect of the art of the endocrine profession. Identification and referral of patients requiring in-hospital endocrine advice is critical for patients, whereas adequate endocrine inpatient case load is essential to provide a comprehensive and contemporary training for future endocrinologists. We are hopeful that this report can generate more interest in current endocrine inpatient care and encourage further studies and reports of existing working models.

## Data Availability

All data and materials used in the writing are described in the manuscript and are available and no additional data exist. The data that support the findings of this study are available from Dr. Esther Osher, but restrictions apply to the availability of these data, which were used under license for the current study, and so are not publicly available. Data are however available from the authors upon reasonable re-quest and with permission of Tel Aviv Sourasky Medical Center Helsinki Committee.
